# Visualizing Cell Type–Specific Glycan Profiles in Tissue Sections With Glycan Painting

**DOI:** 10.21769/BioProtoc.5859

**Published:** 2026-10-20

**Authors:** Akira Nagasaki, Yoichi Shinkai

**Affiliations:** Cellular and Molecular Biotechnology Research Institute, National Institute of Advanced Industrial Science and Technology (AIST), 1-1-1 Higashi, Tsukuba, Ibaraki, Japan

**Keywords:** Glycans, Lectins, Fluorescent staining, Tissue section staining, Multicolor visualization, Glycan painting

## Abstract

Glycans play critical roles in numerous biological processes. Therefore, visualizing the spatial distribution of glycans within cells and tissues is essential for understanding tissue architecture and cellular identity. Glycan painting is a novel tissue-staining method that employs three fluorescently labeled lectins with different glycan-binding specificities to visualize cell type–specific glycan profiles as unique color patterns. In this study, the protocol was validated using formalin-fixed paraffin-embedded sections of mouse embryos. Although Glycan painting is a qualitative method, each cell population that is difficult to distinguish with a single lectin can be differentiated as unique color patterns generated from the combined images of three fluorescent lectins. Moreover, because it does not require costly reagents or specialized equipment, Glycan painting provides a cost-effective and broadly accessible approach for full-color tissue imaging and cell-type discrimination.

Key features

• Glycan painting enables full-color visualization of cell type–specific glycan profiles through simultaneous staining with three fluorescently labeled lectins with different glycan-binding abilities.

• Applicable to formalin-fixed paraffin-embedded tissue sections.

• Provides comprehensive visualization of tissue architecture while simultaneously facilitating cell-type discrimination.

• Compatible with standard fluorescence microscopy and uses readily available lectins and reagents, enabling rapid adoption without specialized instrumentation.

## Graphical overview


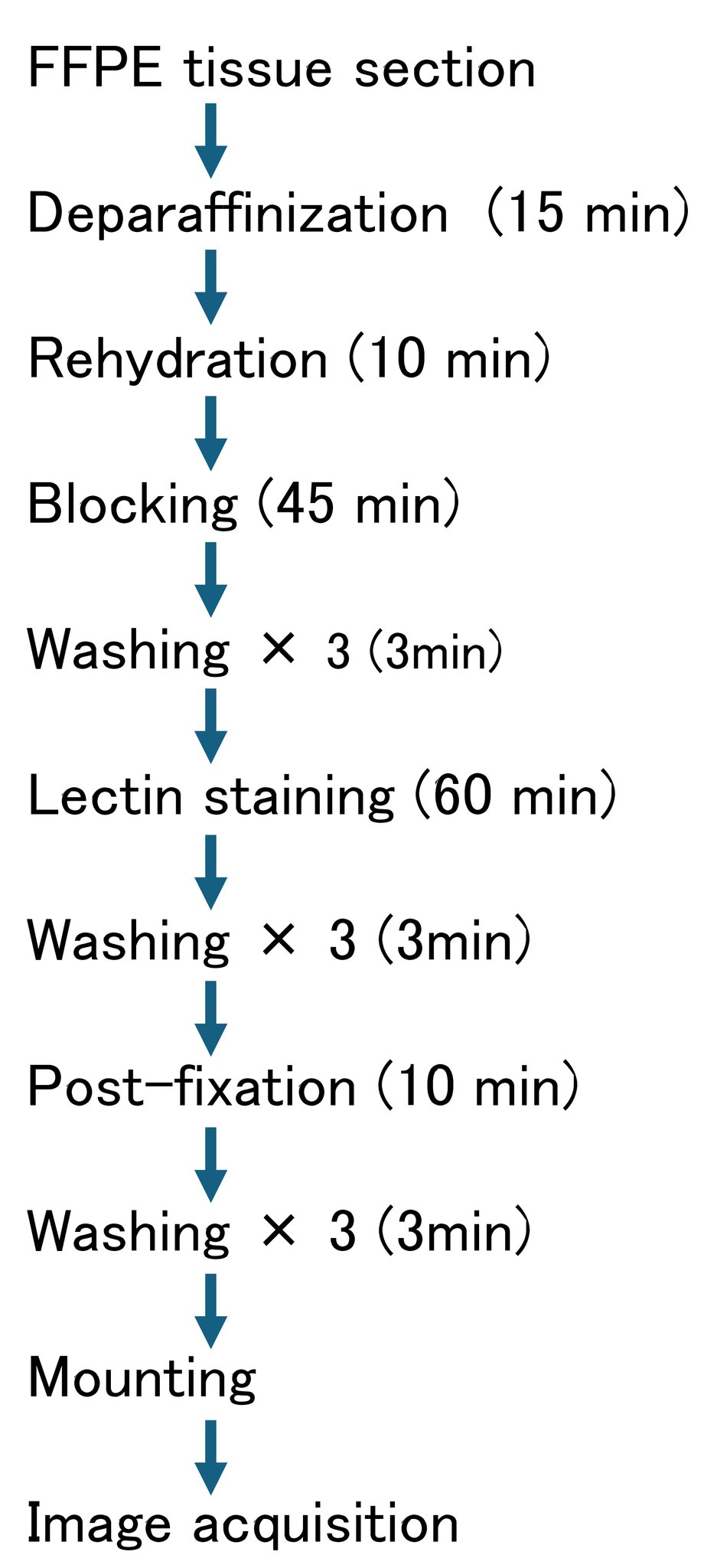


**Graphical overview of the Glycan painting protocol.** The workflow includes deparaffinization and lectin staining. Approximate incubation times are indicated for each step.

## Background

Hematoxylin and eosin (H&E) staining is one of the most fundamental and widely used staining methods and has served as a cornerstone of pathology, histology, and developmental biology for over 100 years [1]. By providing color contrast in otherwise colorless and transparent tissue sections, H&E staining enables visualization of overall tissue architecture and cellular morphology and has long been employed as a fundamental method for histological observation. In H&E staining, the basic dye hematoxylin stains cell nuclei containing RNA and DNA as blue-violet, whereas the acidic dye eosin stains the cytoplasm and extracellular matrix as pink-red. Together, these contrasting dyes reveal a wide range of morphological features, including cell size, nuclear shape, cellular density, and tissue architecture, allowing cell types to be inferred through comprehensive visual assessment.

However, because the identification of cell types in H&E staining relies primarily on morphological features, the ability to distinguish morphologically similar cell populations remains difficult. In addition, H&E staining does not provide direct information regarding cellular function or the state of molecular expression. To address these limitations, immunofluorescent staining using antibodies against cell type–specific proteins has become widely adopted [2]. Immunofluorescent staining enables the visualization of cell type–specific molecular markers, providing information at the molecular expression level that complements conventional morphological observations. While immunofluorescent staining is useful, this staining method also has several limitations, including a restricted multiplex staining capacity, the need to optimize staining conditions, and dependence on predefined molecular markers.

The development of a technology capable of identifying various cell types in biological tissues as distinct color patterns would have broad significance across research fields such as cell and developmental biology, neuroscience, and pathology. In particular, the intuitive visualization of spatial cellular organization and intracellular relationships may provide new perspectives on tissue structure beyond conventional morphology-based analysis. Here, the focus is placed on cell-specific profiles of glycans, which are widely distributed on the cell surface, within cells, and in the extracellular matrix, where they play crucial roles in development and tissue organization [3]. Furthermore, a vast number of glycan-binding molecules, collectively known as lectins, exist throughout the biological world [4]. However, individual glycans do not exhibit strict cell-type specificity, and lectins possess relatively broad and overlapping glycan-binding specificities, limiting their utility as cell identification markers in a conventional single-probe staining approach. In contrast, combinations of glycan profiles exhibit cell-type specificity [5].

We therefore developed a novel staining method termed *glycan painting* in which differences in glycan profiles are translated into distinct color patterns, enabling cell populations to be distinguished based on their glycan composition [6]. In brief, merged images generated from triplex fluorescence staining of tissue sections using different lectins labeled with distinct fluorescent dyes allow different cell populations to be visualized as characteristic colors. In this report, ConA, WGA, and LEL were selected because they recognize distinct glycan structures and provide distinct staining patterns, enabling diverse RGB color combinations for visual discrimination of different tissue and cell populations. This approach is based on the additive mixing of three primary colors of light (red, green, and blue) and is analogous to the color-generation mechanism of an LCD monitor. In this study, glycan painting was applied to comprehensively visualize mouse embryonic tissues by leveraging the broad distribution of glycans and the overlapping glycan-binding specificity of lectins, features traditionally regarded as limitations, to generate full-color tissue images. Further optimization of lectin combinations may improve the performance of glycan painting, depending on the tissue type and biological application.

## Materials and reagents

**Biological materials**

1. Formalin-fixed, paraffin-embedded mouse embryo (E10.5) tissue sections (Genostaff, catalog number: HMPS-02)

2. Concanavalin A, CFR405S conjugate (Fujifilm, catalog number: 559-54431)

3. Wheat germ agglutinin, CFR488A conjugate (Fujifilm, catalog number: 557-54231)

4. Tomato lectin (LEL), Dylight594 conjugate (Thermo Fisher, catalog number: L32471)

*Note: Store all fluorescent lectin conjugates at 4 °C protected from light. Avoid repeated freeze/thaw cycles. In our laboratory, the conjugates retained staining performance for at least six months under these conditions.*

**Reagents**

1. PathoClean (Fujifilm, catalog number: 161-28321)

2. Ethanol (Fujifilm, catalog number: 0596-00486)

3. 20× Tris-buffered saline (TBS), pH 7.4 (Fujifilm, catalog number: 317-90371)

4. D-PBS(+) preparation reagent (Ca, Mg solution) (100×) (Nacalai Tesque, catalog number: 02492-94)

5. 10× carbo-free blocking solution (Vector, catalog number: SP-5040-125)

6. 10% formalin-neutral buffer solution (Fujifilm, catalog number: 062-01661)

7. 1,4-diazabicyclo[2.2.2]octane (DABCO) (Fujifilm, catalog number: 049-25712)

8. Glycerol (Fujifilm, catalog number: 075-00616)

9. Tris(hydroxymethyl)aminomethane (Fujifilm, catalog number: 207-06275)

10. Kaneyonol (Kaneyo soap laundry glue, Kaneyo); if unavailable, 8% polyvinyl alcohol (PVA) (Sigma, catalog number: P8316) or a commercially available mounting medium can be used as an alternative

**Solutions**

1. Wash buffer (see Recipes)

2. Blocking buffer (see Recipes)

3. Staining buffer (see Recipes)

4. Mounting medium (see Recipes)

**Recipes**

*Note: All solutions, except for the mounting medium, are freshly prepared before each experiment and should not be reused.*

**1. Wash buffer (100 mL)**

**Table:** 

Reagent	Final concentration	Volume
20× TBS (pH7.4)	1×	5 mL
Ca, Mg solution (100×)	1×	1 mL
Double-distilled water		94 mL

**2. Blocking buffer (10 mL)**

**Table:** 

Reagent	Final concentration	Volume
20× TBS (pH 7.4)	1×	0.5 mL
Ca, Mg solution (100×)	1×	0.1 mL
10× carbo-free blocking solution	1×	1 mL
Double-distilled water		8.4 mL

**3. Staining buffer (1 mL)**

**Table:** 

Reagent	Final concentration	Volume
20× TBS (pH 7.4)	1×	0.05 mL
Ca, Mg solution (100×)	1×	0.01 mL
Concanavalin A, CFR405S conjugate	2 μg/mL	2 μL
Wheat germ agglutinin, CFR488A conjugate	2 μg/mL	2 μL
Tomato lectin (LEL), DyLight594 conjugate	2 μg/mL	2 μL
Double-distilled water		1 mL

**4. Mounting medium (40 mL)**

**Table:** 

Reagent	Final concentration	Quantity or volume
80% glycerol	16%	8 mL
Kaneyonol or 8% PVA	75%	30 mL
2 M Tris-HCl (pH 8.0)	60 mM	1.2 mL
DABCO	1.5%	0.625 g
Double-distilled water		Up to 40 mL

*Note: Kaneyonol or 8% PVA is used as a viscosity-enhancing agent in the mounting medium to maintain an appropriate layer of mounting medium beneath the coverslip. Upon drying, the mounting medium solidifies and helps stabilize the coverslip. The mounting medium is stored in the dark and reused for subsequent experiments.*

**Laboratory supplies**

1. 1.5 mL microtube (FastGene, catalog number: FG-MCT15A)

2. 50 mL disposable centrifuge tube (Iwaki, catalog number: 2342-050N)

3. 100 mL disposable centrifuge tube (Iwaki, catalog number: 2355-100N)

4. Coverslip (Matsunami, catalog number: C022221)

5. A-PAP pen (Matsunami, catalog number: GMP0020)

## Equipment

1. Confocal microscopy (AX, Nikon, Tokyo, Japan, model: Nikon AX with NSPARC Super-Resolution Confocal Laser Microscope System)

2. Aspirator (IWAKI, AGC Techno Glass, Shizuoka, Japan, catalog number: VPUMP-140)

## Software and datasets

1. Fiji/ImageJ (version 2.16.0, National Institutes of Health (NIH), USA; used for fluorescence image processing, channel merging, RGB analysis, and image quantification

2. NIS-Elements AR (version 6.20, Nikon, Tokyo, Japan); used for image acquisition and processing of confocal microscopy data

## Procedure

A schematic overview of the glycan painting protocol from FFPE tissue section preparation to fluorescence image acquisition is shown in the Graphical overview.

**A. Deparaffinization of FFPE tissue sections**

1. Deparaffinize tissue sections in PathoClean in 50 mL disposable centrifuge tubes, as follows:

a. Immerse sections in PathoClean for 5 min.

b. Transfer sections to fresh PathoClean for 3 min.

c. Transfer sections to fresh PathoClean for 2 min.

d. Transfer sections to fresh PathoClean for 1 min.

2. Rehydrate sections through a graded ethanol series in 50 mL disposable centrifuge tubes, as follows:

a. Immerse sections in 100% ethanol for 5 min.

b. Transfer sections to fresh 100% ethanol for 3 min.

c. Immerse sections in 95% ethanol for 2 min.

3. Rinse slides in double-distilled water and immerse them in TBS buffer in 50 mL disposable centrifuge tubes. The rehydration procedure is performed according to the manufacturer’s recommended protocol for PathoClean (Fujifilm).

**B. Blocking**

1. To prevent tissue sections from drying, gently wipe excess moisture from the glass slide using a laboratory wipe and draw a hydrophobic barrier around the section with an A-PAP pen. Allow the barrier to dry completely for approximately 1 min at room temperature.

2. After the A-PAP pen barrier has dried, add the blocking buffer to the inner area enclosed by the hydrophobic barrier and incubate for 45 min at room temperature.

**C. Lectin staining**

1. Wash the blocked slides three times with washing buffer. During each wash, add washing buffer to tissue sections using a micropipette and carefully remove the buffer with an aspirator.

2. Add 400 μL of staining buffer per 2 cm^2^ of the area enclosed by the hydrophobic barrier pen, ensuring that the tissue section is completely covered. Incubate for 60 min at room temperature in the dark or overnight at 4 °C. To prevent evaporation of the staining buffer during incubation, place the slides in a 10-cm Petri dish or a humidified chamber.

3. Wash the slides three times with approximately 400 μL of washing buffer per 2 cm^2^ of tissue area.

4. Immediately after washing, fix the stained sections with 10% formalin-neutral buffer solution for 10 min at room temperature in the dark. This post-fixation is performed to stabilize lectin–glycan interactions and preserve the fluorescent staining patterns during subsequent processing.

5. Wash slides three times with washing buffer under the same conditions as described above.

**Caution:** 10% formalin-neutral buffer solution is toxic and should be handled in a certified chemical fume hood while wearing appropriate personal protective equipment (gloves, lab coat, and eye protection).

**Pause point:** If necessary, the incubation can be extended overnight at 4 °C in the dark without affecting staining performance.

**Critical:** Do not allow the tissue sections to dry at any stage of the procedure. Drying may increase nonspecific background staining and reduce staining reproducibility.

**Critical:** ConA requires divalent cation for glycan binding. Therefore, the staining buffer should contain Ca^2+^ to ensure optimal staining.

**D. Mounting**

1. Apply an appropriate amount of mounting medium onto the tissue section and carefully place a coverslip over the sections while avoiding the introduction of air bubbles.

2. Allow the slide to incubate for 3 h at room temperature in a dry and dark place.

**Pause point**: Mounted slides may be stored at 4 °C, protected from light, for several days before imaging.

**E. Image acquisition**

For conventional fluorescence microscopy, acquire images using DAPI, GFP, and Texas Red filter sets corresponding to ConA-CFR405S, WGA-CFR488A, and LEL-DyLight 594, respectively. In the case of confocal microscopy, acquire images using the 405, 488, and 561 nm laser lines. Figure 1 shows a composite tiled image generated from 72 confocal images acquired for each fluorescent lectin, together with the corresponding merged image. A 20× objective lens was used to provide a sufficient field of view while maintaining adequate spatial resolution for visualizing and comparing glycan distribution patterns across different tissues and cell populations. Images were acquired at a resolution of 1,024 × 1,024 pixels with two-line averaging and a pixel dwell time of 3.2 μs. The laser power was set to 5.7%, 1.8%, and 10.2% for the 405, 488, and 561 nm lasers, respectively. Detector gain was set to 85, and the pinhole was set to 1.4 Airy units. These settings were kept constant for all images acquired within each experiment. However, laser power, detector gain, and other acquisition parameters were adjusted as needed between samples to obtain optimal image quality while avoiding signal saturation.

**Figure 1.**
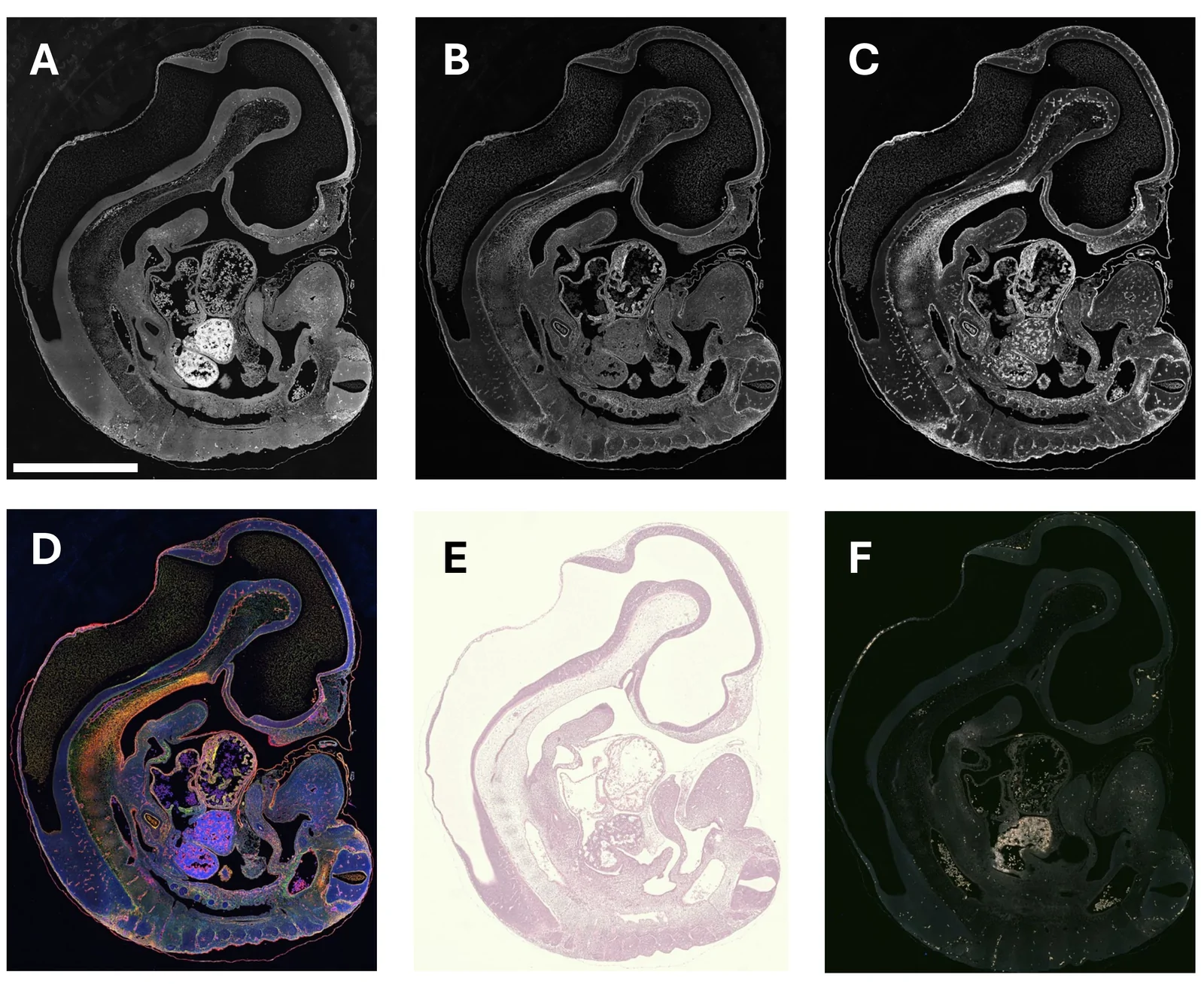
Confocal images of a mouse embryo E10.5 section stained using glycan painting. The mouse embryo section was stained with (A) ConA-CFR405S, (B) WGA-CFR488A, and (C) LEL-DyLight594. (D) Merged image of triple staining. (E) H&E-stained image and (F) autofluorescence images of serial sections prepared from the same FFPE block. Scale bar: 1 mm.

## Data analysis

Merged images were adjusted for brightness and contrast using FIJI (ImageJ). In this protocol, image analysis was primarily qualitative, focusing on comparisons of glycan-dependent color patterns among different cell populations. The representative images in Figure 1 were selected from an FFPE mouse embryo section, because it contains multiple organs and diverse cell populations within a single section, allowing visualization and comparison of characteristic glycan distribution patterns across different tissues and cell types.

Potential spectral overlap and channel cross-talk should be considered when interpreting fluorescence signals. Therefore, glycan painting is intended for qualitative interpretation of relative RGB color patterns, rather than quantitative comparison of individual fluorophore intensities or absolute fluorescence levels.

Quantitative comparison of fluorescence intensities across different experiments or imaging systems requires standardized reference samples for signal normalization. Because such a standardization system is currently under development, this protocol is intended for the qualitative interpretation of characteristic RGB color patterns rather than the quantitative assessment of absolute fluorescence intensities.

## Validation of protocol

This staining protocol was independently repeated in three separate experiments on different days using FFPE mouse embryo sections, yielding reproducible staining results. Representative images from each experiment are shown in Figure 2. The broader applicability of glycan painting has been demonstrated in our previous bioRxiv preprint (Figure 2 from [6]), which described its application to mouse tissue sections at different developmental stages.

**Figure 2.**
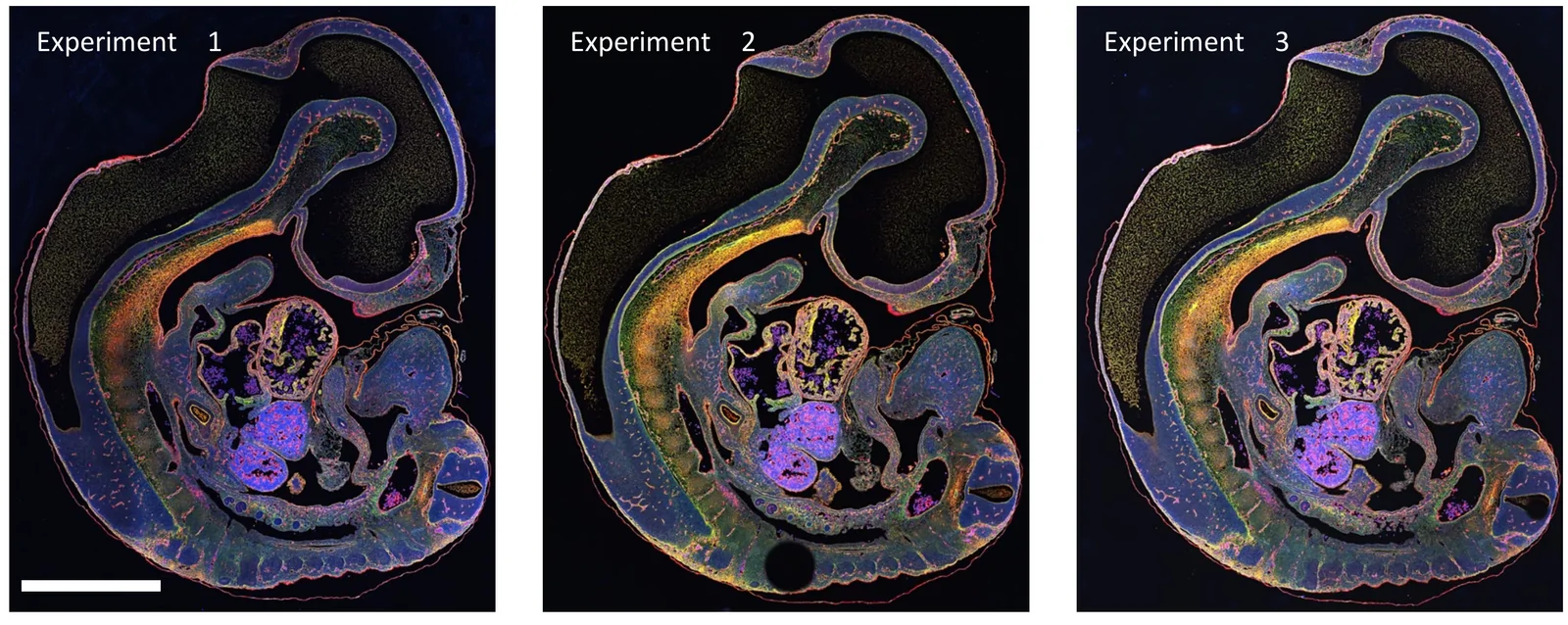
Representative images from three independent staining experiments performed on different days. All images were acquired under identical imaging conditions, and similar fluorescence patterns were reproducibly observed across the three experiments. Scale bar: 1 mm.

## General notes and troubleshooting

**General notes**

1. Tissue sections should not be allowed to dry during staining steps.

2. ConA and certain other lectins require divalent cations, such as Ca^2+^, for optimal glycan binding.

3. Commercial mounting reagents compatible with fluorescence imaging, including PVA-DABCO-based formulations, may be used as alternatives to the mounting medium described above.

4. For imaging of large tissue areas, near-infrared nuclear staining DRAQ5 is recommended for reliable focusing.

5. Carbo-free blocking solution is recommended for blocking before lectin staining to avoid an increase in background fluorescence.

**Troubleshooting**

**Problem 1:** High background fluorescence.

Possible cause: The tissue section has dried during the staining procedure.

Solution: Avoid drying of sections and perform incubation in a humidified chamber.

**Problem 2:** Rapid fading of fluorescence.

Possible cause: Excessive exposure to excitation light.

Solution: Minimize light exposure and use an antifade mounting medium when possible.

**Problem 3:** Uneven staining.

Possible cause: Insufficient reagent coverage on the tissue section.

Solution: Ensure the entire tissue area is completely covered with solution.

## References

[1] Fischer A. H., Jacobson K. A., Rose J. and Zeller R. (2008). Hematoxylin and Eosin Staining of Tissue and Cell Sections. Cold Spring Harb Protoc. 2008(5): pdb.prot4986. https://doi.org/10.1101/pdb.prot4986 21356829

[2] Magaki S., Hojat S. A., Wei B., So A. and Yong W. H. (2019). An introduction to the performance of immunohistochemistry. Methods Mol Biol. 1897: 289 298 298. 10.1007/978-1-4939-8935-5_25 PMC6749998 30539453

[3] Varki A. (2016). Biological roles of glycans. Glycobiology. 27(1): 3 49 49. 10.1093/glycob/cww086 PMC5884436 27558841

[4] Manning J. C., Romero A., Habermann F. A., García Caballero G., Kaltner H. and Gabius H. J. (2016). Lectins: a primer for histochemists and cell biologists. Histochem Cell Biol. 147(2): 199 222 222. 10.1007/s00418-016-1524-6 28013368

[5] Martínez-Carrasco R. and Argüeso P. (2023). Characterization of Cell Surface Glycan Profiles in Human and Mouse Corneas Using Lectin Microarrays. Cells. 12(19): 2356 10.3390/cells12192356 PMC10572028 37830569

[6] Nagasaki A. (2026). Glycan Painting: Triplex Lectin Staining Enables Visualization of Cell-Type-Specific Glycan Profiles in Tissue Sections. bioRxiv. e720979. 10.64898/2026.04.27.720979

